# Evidence-based interventions to restore or improve female fertility in women aged 30–42 years: a systematic review by etiology and evidence level

**DOI:** 10.3389/fendo.2026.1741198

**Published:** 2026-06-09

**Authors:** Maria Jimena Barroso Alverde, Sion Yu, Daniel Pascal Pontón, Denise Niza Benardete Harari, José Elias Tesone Lasman

**Affiliations:** 1 Anahuac University of North Mexico, Huixquilucan de Degollado, Mexico; 2 Avida Fertility, Mexico City, Mexico

**Keywords:** assisted reproductive technology, evidence-based interventions, female infertility, live birth, women aged 30–42

## Abstract

**Importance:**

Female infertility affects approximately one in six couples worldwide and disproportionately impacts women aged 30–42 years, yet evidence on which interventions improve clinically meaningful outcomes (live birth and safety) is heterogeneous.

**Objective:**

The aim of this study was to synthesize evidence on interventions that restore or improve fertility in women aged 30–42 years, grouped by etiology.

**Evidence review:**

We conducted a PRISMA (Preferred Reporting Items for Systematic reviews and Meta-Analyses)-compliant systematic review (PROSPERO CRD420251109428). PubMed and Scopus were searched for English/Spanish studies (1 January 2020 to 31 July 2025). Eligible designs were randomized trials and non-randomized interventional and observational studies. We aligned outcomes to the infertility core outcome set, prioritizing live birth per woman, then ongoing/clinical pregnancy and prespecified safety endpoints. Risk of bias used RoB 2 [randomized controlled trials (RCTs)], ROBINS-I (non-randomized), and JBI (observational). When pooling was not feasible, we applied SwiM (Synthesis Without Meta-analysis) and summarized by etiology and intervention class.

**Findings:**

A total of 21 studies met inclusion criteria for synthesis, and two additional randomized protocols were summarized narratively (23 records tracked overall). Interventions spanned hormonal therapies, assisted reproductive technologies (ART) strategies, surgical procedures, lifestyle and psychosocial programs, and investigational adjuncts. Across etiologies, several interventions were associated with improved pregnancy-related outcomes—such as myo-inositol, clomiphene with sildenafil, selected luteal support regimens, structured stress management, and specific ART strategies—whereas live-birth effects were supported by only moderate-certainty evidence, limited by small samples, heterogeneity, and indirectness. Diagnostic and prognostic approaches (e.g., serum progesterone profiling, BAP-EB receptivity assay, and endometrial T-bet/GATA3 ratio) showed predictive value but no therapeutic effect. Safety reporting was limited; few studies reported ovarian hyperstimulation syndrome, multiple gestation, or neonatal outcomes, and patient-reported outcomes were seldom assessed.

**Conclusions and relevance:**

For women aged 30–42 years, established hormonal and ART strategies improve pregnancy outcomes; however, certainty for live birth and safety remains limited. Clinical care should prioritize standard therapies and shared decision-making, acknowledging evidence gaps. Future trials must center on live birth, adopt standardized core outcomes, and consistently report maternal–neonatal safety.

**Systematic review registration:**

https://www.crd.york.ac.uk/prospero/, identifier CRD420251109428.

## Introduction

Infertility in women of reproductive age is a significant global health issue. It is commonly defined as the failure to achieve pregnancy after 12 months of regular, unprotected intercourse. Recent studies suggest that it affects up to 17.5% of adults worldwide, making it one of the most prevalent conditions in reproductive health (1). In countries such as China, the proportion may be even higher, reflecting notable geographical differences in prevalence (2). Globally, approximately one in six couples is affected, with female factors responsible for nearly half of the cases (3).

The causes of female infertility are multifactorial and frequently interrelated. Ovulatory disorders, particularly polycystic ovary syndrome (PCOS), are among the most common contributors, affecting a notable percentage of reproductive-age women (4). Other etiologies include endometriosis, blocked fallopian tubes, low ovarian reserve, and uterine abnormalities such as adenomyosis (5, 6). These conditions can present independently or concurrently, often making diagnosis and treatment more complex.

Beyond the physical aspects, infertility often results in profound emotional and social consequences. Women coping with this condition frequently experience psychological distress, including anxiety and depression. In many settings, societal expectations around motherhood can intensify feelings of isolation and emotional burden (7). At the same time, the high cost of treatment—particularly assisted reproductive technologies (ART)—can restrict access and widen health disparities (8).

Although treatment options have expanded, research on infertility interventions is often inconsistent and lacks standardization. Studies vary widely in how they define infertility, measure outcomes, and select participants, which makes cross-comparison difficult. In addition, methodological concerns such as retrospective design and unaccounted confounders continue to limit the strength of existing evidence (2, 9). Moreover, there is a lack of comparative data that clearly evaluate which interventions are most effective for different underlying conditions (6).

Such gaps limit the creation of standardized clinical guidelines and present challenges for healthcare providers who must choose among treatment options with variable or unclear evidence (3). There is a clear need for structured and evidence-based approaches that can better inform personalized fertility care.

To improve outcomes, early identification of modifiable risk factors—such as smoking, diet, and obesity—is essential. In addition, clinical markers like age at menarche may offer predictive insights into future fertility issues, aiding in earlier intervention (2).

In response to these challenges, this systematic review aims to critically evaluate the current clinical evidence on interventions intended to restore or improve fertility among reproductive-age women. This includes a wide range of evidence-based options such as hormonal therapies, ART, surgical procedures, and lifestyle modifications. By synthesizing these findings, the review seeks to support more effective and individualized treatment planning. The research question guiding this review is: What evidence-based clinical interventions are effective in restoring or improving fertility among women of reproductive age?

This review focuses on women aged 30–42 years and organizes evidence by etiology (PCOS, endometriosis/adenomyosis, poor ovarian response, unexplained infertility, and recurrent implantation failure), alongside cross-etiology ART strategies. We prioritize live birth per woman randomized and safety, aligning outcomes with the infertility core outcome set.

Findings from this review could help shape future clinical guidelines and highlight key areas for future research, including comparative effectiveness and tailored therapeutic approaches.

## Materials and methods

This systematic review was conducted in accordance with the PRISMA (Preferred Reporting Items for Systematic reviews and Meta-Analyses) 2020 guidelines. The review protocol was registered in the International Prospective Register of Systematic Reviews (PROSPERO) [CRD420251109428].

Studies were selected based on predefined eligibility criteria structured according to the PICOS framework.

### Eligibility criteria

We applied the PICOS framework:

- Population: Women aged 30–42 years diagnosed with infertility of any cause.
- Interventions: Evidence-based interventions aimed at restoring or improving fertility, including but not limited to hormonal therapies (e.g., vitamin D, myo-inositol, clomiphene, gonadotropins, progesterone, letrozole, and tamoxifen), ART [e.g., *in vitro* fertilization (IVF) protocols, embryo transfer strategies, preimplantation genetic testing, and endometrial preparation], surgical procedures (e.g., intrauterine adhesion repair and ovarian tissue autotransplantation), lifestyle or psychosocial interventions (e.g., stress management programs), and other/experimental approaches (e.g., immune profiling with precision treatment, moxibustion, and atosiban infusion).
- Comparators: No comparator was required for inclusion; studies with or without comparison groups were considered.
- Outcomes: The primary outcome was live birth per woman randomized. Secondary outcomes were ovulation, clinical pregnancy (defined as ultrasound confirmation of a gestational sac), ongoing pregnancy (≥12 weeks, or as defined by study), cumulative live birth, and pregnancy loss (miscarriage, ectopic, stillbirth, and termination). Safety outcomes included ovarian hyperstimulation syndrome (OHSS), multiple gestation, preterm birth (<37 weeks), low birth weight (<2,500 g), neonatal mortality (<28 days), and congenital anomalies. Where available, biochemical pregnancy (hCG only) was extracted but not synthesized. Patient-reported outcomes (mental health and quality of life) were also collected.
- Study types: Randomized controlled trials (RCTs), cohort studies, case–control studies, and systematic reviews published in peer-reviewed journals were eligible.
- Language and timeframe: Only studies published in English or Spanish between January 2020 and July 2025 were included.

### Information sources

We searched PubMed and Scopus from 1 January 2020 to 31 July 2025 for studies published in English or Spanish. Because of access limitations, Embase and Web of Science were not searched.

- PubMed: 3,767 records; 616 (2020–2025); 610 (English/Spanish); 219 after applying study-type filters.
- Scopus: 730 records; 296 (English/Spanish).
- Total records identified: 515. After removing 75 duplicates, 440 remained for screening.

### Study selection process

**Figure 1:**
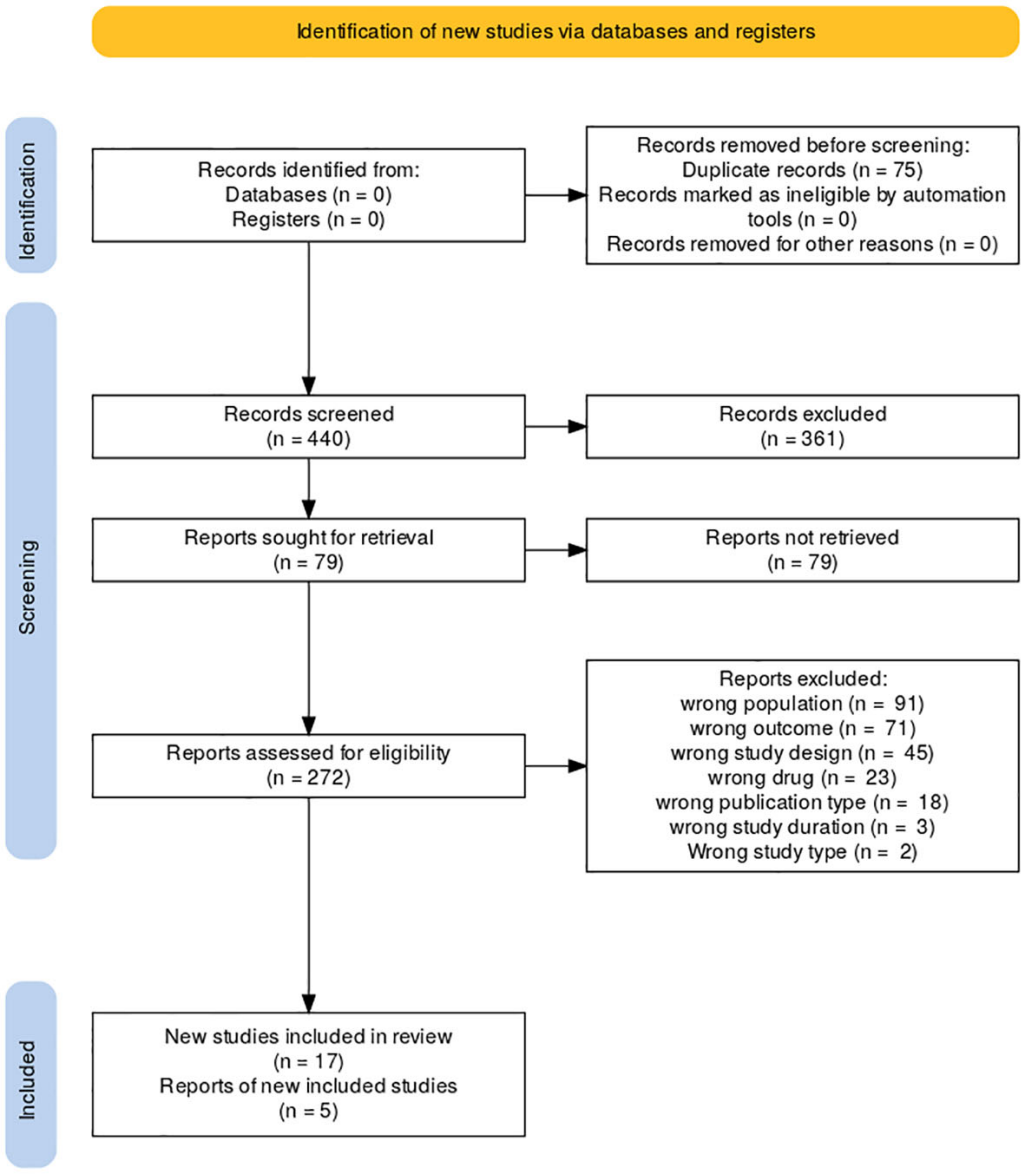
PRISMA 2020 flow diagram illustrating study identification, screening, eligibility assessment, and inclusion process for studies evaluating fertility-restoring or fertility-improving interventions in women aged 30–42 years.

Two reviewers independently screened records (title/abstract, full text); disagreements were resolved by consensus or a third reviewer. Reports not retrieved primarily included records without accessible full texts, conference abstracts without complete manuscripts, duplicate indexed records, and articles unavailable through institutional or open-access sources. Because these reports were not assessed at full-text level, potential retrieval bias cannot be excluded and was acknowledged as a limitation. Screening results are shown in the PRISMA flow diagram (**Figure 1**).

### Data extraction and risk of bias

Data were extracted in duplicate into a standardized form. Risk of bias was assessed using RoB 2 for RCTs, ROBINS-I for non-randomized studies, and the JBI checklist for observational cohorts.

### Data synthesis

We grouped studies by etiology and intervention class. Outcomes were aligned with the infertility core outcome set, prioritizing live birth per woman randomized whenever available. Secondary outcomes included ovulation, biochemical pregnancy, clinical pregnancy, ongoing pregnancy, cumulative live birth, pregnancy loss, safety outcomes, and patient-reported outcomes. Where definitions varied across studies (e.g., ongoing pregnancy ≥10–12 weeks), study definitions were accepted and heterogeneity was documented.

Quantitative meta-analysis was considered but deemed inappropriate because of substantial clinical and methodological heterogeneity across included studies, including differences in infertility etiology, intervention type, ART protocol, comparator group, outcome definition, follow-up duration, and reporting unit. Outcomes were inconsistently reported as per woman, per cycle, per retrieval, or per embryo transfer, which precluded meaningful pooled estimates. In addition, most intervention–outcome comparisons were represented by single studies, preventing statistically valid subgroup meta-analysis.

Therefore, synthesis was performed using the SWiM (Synthesis Without Meta-analysis) framework. Studies were grouped by etiology (PCOS, endometriosis/adenomyosis, poor ovarian response, unexplained infertility, and recurrent implantation failure) and by intervention class (hormonal, ART-related, surgical, lifestyle/psychosocial, and investigational adjuncts). Findings were synthesized narratively and organized according to study design and outcome type.

To avoid overinterpretation across evidence hierarchies, RCTs were summarized separately from observational, retrospective, registry-based, diagnostic, and prognostic evidence.

## Results

### Study selection and overall characteristics

A total of 21 completed studies were included in the evidence synthesis, and two ongoing randomized trial protocols were summarized separately. Although live birth per woman was prespecified as the primary outcome, live birth was not consistently reported across the included studies. Most studies reported surrogate reproductive endpoints, including ovulation, biochemical pregnancy, clinical pregnancy, implantation, ongoing pregnancy, oocyte yield, or embryo-related outcomes. Therefore, results are presented separately for live-birth outcomes and pregnancy-related surrogate outcomes to avoid overstating the strength of the evidence.

Interventions included hormonal, ART, surgical, lifestyle and psychosocial, and investigational adjunct domains.

Most included studies enrolled broader reproductive-age populations that overlapped with the target age range rather than exclusively women aged 30–42 years. Consequently, indirectness was incorporated into certainty assessments, and age-specific subgroup analysis was not feasible because most studies did not report stratified outcomes for women aged 30–42 years.

**Table 1** provides detailed characteristics of the 21 included interventional studies, including design, population, interventions, comparators, outcomes, and key findings.

**Table 1:** Characteristics of included interventional studies (*n* = 21).

Author (year)	Etiology	Design	*N* (age)	Intervention	Comparator	Primary outcomes (unit)	Key results
Badihi (2025)	RIF	RCT	Women with RIF, 18–40	Vitamin D 50,000 IU weekly + vaginal probiotics	Standard IVF	Clinical pregnancy; live birth (per woman)	↑ clinical pregnancy; live-birth signal
Bezerra Espínola (2021)	Cross-etiology ART	RCT	≤42 (mean 35–36)	Vitamin D3 + myo-inositol + folic acid + melatonin	Standard IVF	Implantation, clinical pregnancy, ongoing pregnancy	↑ implantation and pregnancy; no live-birth data
Doryanizadeh (2021)	Cross-etiology ART	RCT	20–40 (mean 32)	Calcitriol 0.5 μg daily × 4 weeks	Placebo	Biochemical, clinical pregnancy	↑ biochemical pregnancy (sig); ↑ clinical NS; no live-birth data
Ebrahimi (2023)	Cross-etiology ART (FET prep)	RCT	18–42 (mean 30–31)	Tamoxifen + gonadotropin	Estradiol + GnRH agonist (HRT)	Chemical, clinical, ongoing pregnancy	Comparable to HRT; no live-birth data
Hu (2024)	Cross-etiology ART	RCT	20–37	PGT-A	Conventional IVF	CLBR (per woman), pregnancy loss	No overall CLBR benefit; ↓ loss in low-oocyte group; subgroup neonatal BW differences
Ishizuka (2021)	POI (surgical/hormonal)	Cohort	22–46 (mean 35.8)	HRT ± ovarian stimulation	No stimulation	Live birth, clinical pregnancy	Live birth 10.7%
Koumparou (2021)	Psychosocial	RCT	≤42	8-week stress program (biofeedback, relaxation, CBT)	Standard IVF	Stress/anxiety scores; clinical pregnancy	↓ stress/anxiety; exploratory pregnancy ↑; no live-birth data
Labarta (2021)	Prognostic (FET)	Cohort	18–42 (mean 38)	Serum mid-luteal progesterone profiling	None	Ongoing pregnancy (per transfer)	Higher mid-luteal P associated with ongoing pregnancy
Lédeé (2025)	RIF	RCT	≤38 (mean 33.3)	Immune profiling + precision therapy (glucocorticoids, intralipids, HCG, scratching)	Conventional ET	Live birth (per woman)	↑ live birth
Lee (2023)	Prognostic	Cohort	≥35	BAP-EB assay	None	CLBR (per woman)	Modest prediction (AUC ~0.61)
Li (2022)	Prognostic	Retrospective	≤45 (median 32–33)	T-bet/GATA3 ratio	None	Live birth (per woman)	High ratio independently ↓ live birth (OR 0.28, cutoff 0.22)
Libby (2021)	Registry safety	Database	IVF patients (mean 34–35)	Fresh autologous IVF	Sterilized vs. infertile cohorts	Live birth; neonatal outcomes	Obstetric/neonatal outcomes similar; registry only, no efficacy inference
Moens (2025)	Cross-etiology ART	Prospective cohort	IVF/ICSI, mean 36.3 (19–48)	Freeze-all policy	Fresh transfer	Ongoing pregnancy; cumulative pregnancy	↑ ongoing pregnancy with freeze-all
Mohammadi (2021)	POR	RCT	20–43 (mean 35–36)	Myo-inositol 4 g + folic acid × 12 weeks	Folic acid alone	Fertilization, OSI, biochemical/clinical pregnancy	↑ fertilization and pregnancy; no live-birth data
Niu (2023)	Cross-etiology ART (luteal support)	RCT	20–40 (mean 31)	Oral progesterone 400 or 600 mg/day	Vaginal progesterone 90 mg/day	Ongoing pregnancy, live birth (per woman)	400 mg non-inferior; 600 mg not; live birth NS
Sarhan (2023)	Unexplained infertility	RCT	18–40 (median 32)	Clomiphene + sildenafil 20 mg BID	Clomiphene + placebo	Clinical, chemical pregnancy; ovulation; endometrial thickness	↑ thickness and clinical pregnancy; no live-birth data
Shapira (2020)	POR (surgical)	Multicenter cohort	21–45 (mean 32.7)	OT autotransplantation ± IVF	None	Pregnancy, live birth (per woman)	50 pregnancies, 44 live births (~42%); miscarriage 6%
Song (2024)	POR	RCT	35–44 (POSEIDON 2a)	RN8 moxibustion + IVF	IVF alone	Oocytes, embryos, CLBR (per retrieval)	↑ oocyte yield; CLBR 27.3% vs. 13.3%
Tang (2022)	RIF	RCT	<40 (median 33)	IV atosiban 6.75 mg bolus pre-ET	Placebo	Live birth, clinical pregnancy (per woman)	Live birth NS; some intermediate signals
Tehraninejad (2024)	Cross-etiology ART (luteal support)	RCT	18–42 (mean 35–36)	Vaginal vs. SC vs. IM progesterone	Comparison across routes	Chemical, clinical, ongoing pregnancy	IM ↑ chemical pregnancy only; ongoing/live birth NS
Carpinello (2021)	Unexplained infertility (IUI)	Retrospective cohort	18–44 (mean 34)	OS-IUI (clomiphene/letrozole)	Natural cycle IUI	Clinical, ongoing pregnancy; multiple gestation	Modestly higher pregnancy rates; increased multiple gestation

### Higher-certainty interventional evidence from randomized controlled trials

In PCOS and other ovulatory disorders, randomized trials of myo-inositol with folic acid pre-treatment and clomiphene combined with sildenafil were associated with improvements in intermediate reproductive outcomes, including fertilization, endometrial thickness, and clinical pregnancy, although live-birth outcomes were not reported.

In recurrent implantation failure, three adjunctive strategies were evaluated. A small randomized trial of vitamin D plus vaginal probiotics increased clinical pregnancy and showed a numerically higher live-birth outcomes, though precision was limited. According to Badihi et al., in women with recurrent implantation failure, combined vaginal probiotics and vitamin D supplementation resulted in higher pregnancy rates than probiotics alone, vitamin D alone, or standard IVF treatment (46.4% vs. 14.2%, 17.8%, and 10.7%, respectively).

A double-blind randomized trial of atosiban given prior to embryo transfer did not significantly increase live birth rates compared with placebo despite signals in intermediate outcomes. In an RCT by Lédeé, they evaluated endometrial immune profiling with precision therapy; live-birth rates were significantly higher with personalized care compared with conventional embryo transfer approaches [41.4% vs. 29.7%; odds ratio (OR) 1.68, 95% confidence interval (CI) 1.04–2.73]. Benefits were especially notable among patients with ≥2 prior failed embryo transfers (48.1% vs. 23.4%; OR 3.03, 95% CI 1.17–7.85).

ART strategies evaluated across etiologies included preimplantation genetic testing for aneuploidy, embryo-transfer policies, and luteal-support regimens. A large multicenter randomized trial by Hu et al. of preimplantation genetic testing for aneuploidy (PGT-A) did not demonstrate an overall cumulative live-birth advantage compared with conventional IVF-ET. Among women with 19–23 retrieved oocytes, cumulative live-birth rates were lower in the PGT-A group than in the conventional IVF-ET group (75.6% vs. 87.1%; RR 0.868, 95% CI 0.774–0.973). However, among women with <15 retrieved oocytes, PGT-A was associated with lower cumulative clinical pregnancy loss rates (4.5% vs. 15.0%) without improvement in cumulative live birth.

In a multicenter non-inferiority randomized trial by Niu et al., oral micronized progesterone 400 mg/day demonstrated ongoing pregnancy rates comparable to vaginal progesterone gel (35.3% vs. 38.0%), supporting non-inferiority for luteal phase support in fresh embryo transfer cycles.

Lifestyle and psychosocial interventions were represented by a pilot randomized trial of an 8-week structured stress-management program that improved stress, anxiety, and depression scores, with exploratory associations with higher pregnancy rates. Live-birth outcomes were not reported.

In women with unexpected poor ovarian response meeting POSEIDON Group 2a criteria, Shen Que moxibustion combined with IVF significantly increased retrieved oocytes (5.8 vs. 4.7, *p* = 0.012), mature oocytes (5.0 vs. 3.0, *p* = 0.008), and available embryos (4.0 vs. 2.0, *p* = 0.014). Although cumulative live-birth rates were numerically higher with moxibustion (27.3% vs. 13.3%), statistical significance was not reached.

In endometriosis and adenomyosis, no new interventional trials specifically targeting these etiologies in women aged 30–42 years were identified during the review period.

Overall, randomized evidence suggested favorable effects of selected hormonal, ART-related, and adjunctive interventions on pregnancy-related outcomes, although live-birth reporting remained inconsistent across studies.

### Supporting observational and retrospective evidence

A multicenter cohort of ovarian tissue autotransplantation reported restoration of menses in most patients, 50 pregnancies, and 44 live births across women aged 21–45.

A prospective cohort comparing freeze-all with fresh transfer found higher ongoing pregnancy with freeze-all.

A retrospective donor-insemination cohort reported modestly higher pregnancy rates with ovarian stimulation but increased multiple gestation compared with natural-cycle intrauterine insemination (IUI) (10.8% vs. 2.4%).

A registry-based analysis comparing obstetric and neonatal outcomes in sterilized versus infertile IVF patients reported broadly comparable neonatal outcomes between groups but did not evaluate treatment efficacy.

Surgical fertility restoration beyond ovarian tissue autotransplantation included a long-term cohort of premature ovarian insufficiency managed with hormone therapy with or without ovarian stimulation, although live-birth rates remained low.

Overall, observational evidence suggested possible benefit for selected ART and fertility-preservation strategies, although interpretation remained limited by confounding, non-randomized designs, and heterogeneous outcome reporting.

### Diagnostic and prognostic evidence

Diagnostic and prognostic studies included mid-luteal serum progesterone profiling after frozen embryo transfer, as described by Labarta et al. (2021), an endometrial receptivity assay with modest discrimination for cumulative live birth in women aged 35 years and older reported by Lee et al. (2023), and an endometrial T-bet/GATA3 ratio associated with live-birth probability identified by Li et al. (2022). As these were prognostic rather than therapeutic, they were not considered evidence of intervention benefit.

An observational cohort reported that mid-luteal serum progesterone profiles after frozen embryo transfer were associated with ongoing pregnancy outcomes.

### Ongoing randomized trial protocols

In addition to completed studies, two ongoing randomized protocols were identified, evaluating letrozole plus FSH stimulation and an extracellular matrix patch for intrauterine adhesions. **Supplementary Table 2** presents their designs and planned outcomes.

### Safety and patient-reported outcomes

Safety outcomes were inconsistently captured across included studies. Only a minority reported OHSS, multiple gestation, or neonatal outcomes. Multiple gestation occurred more frequently with ovarian stimulation than natural-cycle IUI (10.8% vs. 2.4%). Freeze-all policies were frequently adopted because of perceived OHSS risk, although detailed OHSS rates were rarely quantified. Neonatal outcomes were mainly available from registry-level data and appeared broadly comparable across groups. Patient-reported outcomes were uncommon and were primarily evaluated in the stress-management trial.

### Certainty of evidence

Certainty of evidence was assessed using the GRADE framework, considering risk of bias, inconsistency, indirectness, imprecision, and publication bias. Evidence for pregnancy-related outcomes was generally rated as moderate because several randomized trials showed favorable direction of effect, but certainty was reduced by heterogeneity in interventions and outcome definitions. Evidence for live birth was rated as low to moderate because live birth was inconsistently reported, several studies were underpowered for this endpoint, and many populations were not restricted to women aged 30–42 years. Evidence for safety outcomes was rated as low because maternal and neonatal adverse outcomes were sparsely and inconsistently reported.

**Table 2** summarizes the risk of bias assessments for randomized, non-randomized, and observational studies.

**Table 2:** Risk of bias summary.

Study	Design	Tool	Overall RoB judgment
Badihi 2025	RCT	RoB 2	Low
Bezerra 2021	RCT	RoB 2	Some concerns
Doryanizadeh 2021	RCT	RoB 2	Low
Ebrahimi 2023	RCT	RoB 2	Some concerns
Hu 2024	RCT	RoB 2	Low
Koumparou 2021	RCT	RoB 2	Some concerns
Lédeé 2025	RCT	RoB 2	Low
Mohammadi 2021	RCT	RoB 2	Some concerns
Niu 2023	RCT	RoB 2	Low
Sarhan 2023	RCT	RoB 2	Some concerns
Song 2024	RCT	RoB 2	Low
Tang 2022	RCT	RoB 2	Low
Tehraninejad 2024	RCT	RoB 2	Some concerns
Ishizuka 2021	Cohort	JBI	Moderate quality
Labarta 2021	Cohort	JBI	Moderate quality
Lee 2023	Cohort	JBI	Moderate quality
Li 2022	Retrospective	JBI	Moderate quality
Libby 2021	Registry	ROBINS-I	Serious risk of bias
Moens 2025	Cohort	JBI	Moderate quality
Shapira 2020	Cohort	JBI	Moderate quality
Carpinello 2021	Retrospective	ROBINS-I	Serious risk of bias

## Discussion

This systematic review synthesized recent evidence on fertility-restoring or fertility-improving interventions in women aged 30–42 years, organized by etiology and evidence level. The main finding is that several hormonal, ART-related, and adjunctive strategies were associated with improvements in pregnancy-related outcomes; however, evidence demonstrating clear improvement in live birth remained limited. This distinction is clinically important because many included studies relied on surrogate endpoints such as biochemical pregnancy, clinical pregnancy, implantation, oocyte yield, or ongoing pregnancy rather than live birth per woman.

Some of the most consistent findings involved ART-related strategies. Non-inferiority of oral versus vaginal progesterone for ongoing pregnancy at specific doses may inform patient-centered luteal-support choices. Preimplantation genetic testing showed prognosis-dependent effects rather than uniform benefit, aligning with caution in guideline recommendations. Freeze-all strategies were associated with higher ongoing pregnancy in observational cohorts but require randomized confirmation to address confounding by indication. Adjunctive approaches for poor ovarian response and recurrent implantation failure—such as moxibustion, immune-guided regimens, vitamin D with probiotics, and atosiban—produced mixed results in small trials and remain investigational pending larger, confirmatory studies with live-birth endpoints. Lifestyle or psychosocial interventions showed promise for mental-health outcomes but lacked robust reproductive endpoints (**Table 3**).

**Table 3:** GRADE certainty summary.

Outcome	Studies	Starting point	Downgrades	Certainty	Rationale
Ovulation	2 RCTs + 1 cohort	High (RCT)	Imprecision (small *N*)	Moderate	Small sample sizes; consistent direction
Pregnancy (any type)	~19 studies (mostly RCTs)	High	Inconsistency (heterogeneous interventions)	Moderate	Wide variation across interventions
Live birth	12 studies (RCTs + cohorts)	High	Imprecision (small pilot RCTs, obs mixed in); Indirectness (age/unit issues)	Low-moderate	Not all robust RCTs; per-cycle units common
Cumulative live birth	3 RCTs	High	Imprecision (few studies)	Moderate	Limited but high-quality
Miscarriage/pregnancy loss	7 RCTs	High	Imprecision (small events, secondary)	Moderate	Secondary outcomes, low event counts
Multiple gestation	2 studies (1 RCT, 1 cohort)	High/Low mix	Imprecision + RoB	Low	Sparse data; rarely primary
Preterm birth/LBW	1 registry cohort	Low	RoB, indirectness	Low	Only registry data available
Oocyte/embryo outcomes	1 RCT	High	Imprecision (single study)	Low	Single small trial
Mental health (PROs)	1 RCT	High	Indirectness (not fertility outcome) + Imprecision	Low	Not generalizable to fertility restoration

### Comparison with prior review/guidelines

In the UK, NICE’s foundational fertility guideline (CG156) set the template for standardized assessment and treatment, and its ongoing full update explicitly prioritizes live birth as a critical outcome—matching our review’s emphasis on live-birth-centered endpoints and safety reporting in women aged 30–42 years. The update is in consultation with an expected publication date of 19 March 2026, and draft evidence documents confirm live birth and clinical pregnancy as primary outcomes for decision-making. Our conclusions—supporting established core treatments while discouraging routine use of unproven adjuncts—align with this direction and with the UK regulator’s cautious stance on “add-ons” (10–13).

The World Health Organization (WHO) frames the clinical evidence within a global access problem: ~17.5% (≈1 in 6) of adults experience infertility, yet affordability, availability, and quality of care remain inconsistent. These data contextualize our finding that high-certainty live-birth evidence is still limited across many interventions and that safety outcomes are underreported—gaps that are particularly consequential for time-sensitive care in women aged 30–42 years (14, 15).

American Society for Reproductive Medicine (ASRM) guidance converges with our results across several domains. For unexplained infertility, ASRM recommends ovarian stimulation with IUI (OS-IUI) as first-line rather than immediate IVF and warns about iatrogenic multiple gestation with stimulation—precisely mirroring our synthesis in which OS-IUI modestly improved pregnancy but increased multiples (16, 17). On ovarian reserve testing, ASRM advises using anti-Müllerian hormone (AMH)/antral follicle count (AFC) to tailor stimulation and predict response rather than as deterministic predictors of spontaneous fertility; our included studies similarly support a prognostic (not therapeutic) role for these measures (18). For PGT-A, ASRM’s 2024 committee opinion concludes that there is no universal live-birth benefit, though aneuploidy-related loss may be reduced in selected contexts—consistent with the large RCT we included that showed no overall cumulative live-birth advantage but subgroup signals (19, 20). On OHSS prevention, ASRM endorses antagonist protocols, individualized triggers, and risk-mitigation strategies; our data indicate that these safer choices do not compromise pregnancy outcomes in this age band (21). Finally, ASRM’s 2024 fertility-drugs-and-cancer guideline finds no conclusive association between fertility drugs and uterine cancer after accounting for underlying infertility—supporting the cautious but reassuring interpretation of limited safety signals in our corpus (22).

Earlier international guidance and reviews reinforce restraint with IVF “add-ons”. ESHRE’s working group and the UK HFEA’s traffic-light system advise against routine use of most adjuncts outside research settings. This squares with our grading of low-to-moderate certainty for adjuncts (e.g., atosiban before ET, vitamin D/probiotics, immune-guided regimens, and moxibustion), where live-birth endpoints remain scarce or imprecise (23, 24).

Overall, our synthesis supports the core positions of NICE, WHO, and ASRM: prioritize established, evidence-based treatments; individualize ART decisions using prognostic (not deterministic) markers; and avoid routine adoption of add-ons without proven live-birth benefit or clear safety profiles. Our review adds 2020–2025, etiology-stratified evidence focused on women aged 30–42 years and highlights persistent deficits in live-birth and maternal–neonatal safety reporting—specific gaps that current guideline updates (e.g., NICE’s in-progress revision) and future trials should address (13).

The overall risk of bias across studies was variable. Most RCTs were judged at low to moderate risk of bias, which strengthens confidence in outcomes such as ovulation and pregnancy. However, several limitations reduced certainty. First, many trials were small pilot studies, leading to imprecision in effect estimates, particularly for live birth and miscarriage outcomes. Second, selective reporting was common, with pregnancy often reported as primary outcome while safety endpoints, mental health outcomes, and laboratory markers were inconsistently assessed. This selective outcome reporting likely inflates effect estimates and limits comparability across studies. Third, observational studies contributed data for outcomes such as neonatal health and mental health, which increased concerns regarding risk of bias, indirectness, and confounding.

This review has limitations. Several included studies enrolled broader reproductive-age populations and did not provide age-specific results for women aged 30–42 years, introducing indirectness into the evidence synthesis and preventing age-specific subgroup analysis. Many studies prioritized surrogate reproductive outcomes rather than live birth and were underpowered for maternal, neonatal, or safety endpoints. Observational and retrospective designs also contributed to potential confounding and selection bias. In addition, the high number of reports not retrieved may have introduced retrieval bias if unavailable studies differed systematically from included studies. Despite these limitations, strengths of this review include a prespecified PROSPERO-registered protocol, use of validated risk-of-bias tools, alignment with the infertility core outcome set, and an etiology-based synthesis focused on women within the advanced reproductive-age range.

Future research should prioritize adequately powered randomized trials that use live birth per woman as the primary endpoint and systematically capture maternal–neonatal safety, including OHSS, multiple gestation, preterm birth, low birth weight, congenital anomalies, and neonatal mortality. Trials should predefine age-restricted analyses for 30–42 years to reduce indirectness, adopt core outcome sets to minimize selective reporting, and integrate validated patient-reported outcomes. Etiology-specific comparative-effectiveness studies are needed to clarify optimal strategies for poor ovarian response, recurrent implantation failure, and unexplained infertility.

## Conclusion and relevance

Across 21 studies in women aged 30–42 years, several interventions, particularly core hormonal regimens and ART strategies, were associated with improvements in pregnancy-related endpoints; however, translation to higher live-birth rates was supported by only moderate-certainty evidence, and safety reporting was sparse. Adjunctive approaches (e.g., immune-guided protocols, vitamin D with probiotics, atosiban, and moxibustion) yielded mixed results in small trials and should be considered investigational until replicated with live-birth-centered designs. Diagnostic and prognostic tools (e.g., mid-luteal progesterone profiling and endometrial assays) may refine prognosis but do not currently constitute therapeutic benefit. For women aged 30–42 years, clinical care should prioritize established, guideline-concordant treatments and shared decision-making that explicitly weighs uncertainty about live birth, multiple gestation risk, and maternal–neonatal outcomes. Future trials must prespecify age-restricted analyses for this population, power for live birth per woman, adopt core outcome sets, and systematically capture maternal–neonatal safety and patient-reported outcomes. High-quality, etiology-specific comparative-effectiveness research, especially for poor ovarian response, recurrent implantation failure, and unexplained infertility, is essential to move from improvements in surrogate pregnancy measures to confident, patient-centered gains in live birth and safety.

## Data Availability

The raw data supporting the conclusions of this article will be made available by the authors, without undue reservation.

## Supplementary material

Supplementary Table 1 summarizes the diagnostic and prognostic studies identified, which were excluded from the main synthesis of interventional evidence.

Supplementary Table 2 presents their designs and planned outcomes.

Supplementary Table 3 provides detailed reporting of safety and patient-reported outcomes, highlighting the inconsistent capture of OHSS, multiples, neonatal endpoints, and psychosocial effects.
