# Supplementary material for: Evidence-based interventions to restore or improve female fertility in women aged 30–42 years: a systematic review by etiology and evidence level
Source: Front Endocrinol (Lausanne). 2026 Jun 9;17:1741198. doi: 10.3389/fendo.2026.1741198 (PMC13286824; doi:10.3389/fendo.2026.1741198)
Supplement: Supplementary Table 1 — summarizes the diagnostic and prognostic studies identified, which were excluded from the main synthesis of interventional evidence. [file DataSheet1.pdf]

Supplementary Material

Table 1 summarizes the diagnostic and prognostic studies identified, which were excluded from the main synthesis of interventional evidence.

Supplementary Table S1

Author (Year)	Marker/test	Population	Outcome predicted	Findings	Note
Labarta (2021)	Mid-luteal serum progesterone profile	Women 18–42 (mean 38), FET	Ongoing pregnancy	Higher mid-luteal P predicted higher ongoing pregnancy	Prognostic only
Lee (2023)	BAP-EB endometrial assay	Women ≥35 undergoing IVF	Cumulative live birth	Modest AUC (~0.61) for predicting CLBR	Prognostic only
Li (2022)	Endometrial T-bet/GATA3 ratio	IVF women ≤45 (median 32–33)	Live birth	High ratio independently predicted lower live birth (cut-off 0.22)	Prognostic only

Table 2 presents their designs and planned outcomes.

Supplementary Table S2

Author (Year)	Population	Intervention	Comparator	Planned outcomes

Guo (2022)	IVF women <42	Letrozole + FSH stimulation	FSH alone	Live birth, pregnancy outcomes
Pang (2022)	Women with intrauterine adhesions ≤40	ECM biological patch + intrauterine balloon after adhesiolysis	Balloon only	Adhesion repair, clinical pregnancy, endometrial thickness

Table 3 provides detailed reporting of safety and patient-reported outcomes, highlighting the inconsistent capture of OHSS, multiples, neonatal endpoints, and psychosocial effects.

Supplementary Table S3

Study	Setting/ intervention	OHSS	Multiple gestation	Preterm birth	LBW	Neonatal death	PROs	Notes
Bezerra 2021	IVF; Vit D3+MI+FA+melatonin	NR	Twins 25% vs 33%	NR	NR	NR	–	Small RCT
Carpinello 2021	Donor IUI OS vs NC	0	↑ multiples: 10.8% vs 2.4%	NR	NR	NR	–	All twins
Hu 2024	IVF; PGT-A	NR	NR	BW lower in	Reported	Reported	–	Neonatal definition: “good

	vs conv IVF			PGT-A group				birth" ≥37 wks, 2500–4000 g
Moens 2025	IVF; freeze-all vs fresh	Freeze-all often for OHSS risk; rates NR	NR	NR	NR	NR	–	Prospective cohort
Niu 2023	IVF; oral vs vaginal progesterone	NR	NR	Perinatal outcomes comparable	Comparable	Comparable	–	Robust definitions
Labarta 2021	FET progesterone profile	NR	NR	NR	NR	NR	–	Miscarriage 19.3% biochemical, 15.5% clinical
Song 2024	POR; moxibustion + IVF	0	NR	NR	NR	NR	–	AEs monitored, rare

Tehraninejad 2024	IVF; progesterone routes	NR	NS	NR	NR	NR	–	Miscarriage NS
Tang 2022	RIF; atosiban	NR	NR	NR	NR	NR	–	Miscarriage NS
Shapira 2020	OT autotransplant	NR	NR	1 preterm at 32 wks	NR	NR	–	Miscarriage ~6%
Libby 2021	Registry IVF	NR	NR	Similar	Similar	Similar	–	Registry; context only
Koumparou 2021	Stress mgmt program	–	–	–	–	–	↓ stress, anxiety, depression	PROs only; no obstetric safety
